# Cognitive Profile of Students Who Enter Higher Education with an Indication of Dyslexia

**DOI:** 10.1371/journal.pone.0038081

**Published:** 2012-06-13

**Authors:** Maaike Callens, Wim Tops, Marc Brysbaert

**Affiliations:** Department of Experimental Psychology, University of Ghent, Ghent, Belgium; University of Leicester, United Kingdom

## Abstract

For languages other than English there is a lack of empirical evidence about the cognitive profile of students entering higher education with a diagnosis of dyslexia. To obtain such evidence, we compared a group of 100 Dutch-speaking students diagnosed with dyslexia with a control group of 100 students without learning disabilities. Our study showed selective deficits in reading and writing (effect sizes for accuracy between d = 1 and d = 2), arithmetic (d≈1), and phonological processing (d>0.7). Except for spelling, these deficits were larger for speed related measures than for accuracy related measures. Students with dyslexia also performed slightly inferior on the KAIT tests of crystallized intelligence, due to the retrieval of verbal information from long-term memory. No significant differences were observed in the KAIT tests of fluid intelligence. The profile we obtained agrees with a recent meta-analysis of English findings suggesting that it generalizes to all alphabetic languages. Implications for special arrangements for students with dyslexia in higher education are outlined.

## Introduction

An increasing number of students with dyslexia enter higher education, most likely due to better assessment, guidance and remediation in primary and secondary education [1]–[2]. This creates a need for information about the characteristics of these students and the best ways to support them. Higher education differs significantly from primary and secondary school. At this age education is no longer compulsory and students have a much wider range of options (certainly compared to primary education, which in most countries is inclusive, with nearly all children given the same curriculum). Therefore, dyslexic students entering higher education can be expected to be a select group, with better than average coping skills and possibly less comorbidity (for the issue of comorbidity in dyslexia, see [3]–[5]).

Still, there is a need for scientific evidence about the cognitive profile of students with dyslexia in higher education, particularly for non-English speaking countries. There are a number of manuals about adult dyslexia and dyslexia in higher education (e.g. [6]–[8]), which contain valuable information for students with dyslexia and their counselors and tutors, but they mainly base their information and recommendations on clinical and educational practice and they focus on the state of affairs in English-speaking countries.

Because of the scarcity of scientific data, at present there are no generally-accepted guidelines, regulations, and standards for compensatory measures. Instead, the clinical experience of the local office of disability services and their considerations tend to prevail [9]. As a result, the special arrangements differ between institutes and are not appreciated by all lecturers. In the absence of theoretical and empirical evidence for the efficacy of such measures lecturers fear that reading disabled students could be beneficiaries of needless exceptions, which create extra work and may be unfair to the other students. Exceptionally, some even doubt whether students with a diagnosis of dyslexia belong in higher education, questioning their cognitive skills and work attitude. Given the current situation, these reactions are not completely without grounds. Sparks and Lovett [9]–[10], for instance, found that offices of disability services in American colleges often give learning disability certificates without empirical justification, and that these certificates tend to be popular when they are linked to course exemptions in colleges with foreign-language requirements.

In the present paper we first discuss what is known about the cognitive profile of students with dyslexia in American and British higher education. Then, we discuss the reasons why generalization to other countries is not straightforward, and we present the data of a new study addressing the limitation.

### The Cognitive Profile of Students with Dyslexia in Higher Education: Evidence from English

A first series of studies in the 1990s [11]–[13] addressed the question whether individuals with dyslexia continued to have problems with reading and spelling in adulthood, or whether remediation, teaching and reading practice in primary and secondary education were able to bridge the initial gap. They had a strong focus on reading and spelling and did not take into account other functions such as memory, attention, planning, and organization. These studies all came to the conclusion that dyslexia is an enduring problem with remaining suboptimal performance for reading and writing in dyslexic university students.

A particularly interesting study was published in the UK by Hatcher, Snowling, and Griffiths [14], because it investigated a broader range of skills. The authors compared the cognitive skills of 23 university students with dyslexia and 50 controls matched on verbal and non-verbal abilities. Participants completed 17 tasks assessing literacy (reading and writing), processing skills (perceptual speed, memory span, and arithmetic), phonological skills (spoonerisms and rapid naming), verbal fluency, verbal abilities (vocabulary test), non-verbal abilities (Raven matrices), and self-reported problems in attention and organization. Surprisingly, the students with dyslexia performed worse on all but the two tasks of general cognitive abilities (Wechsler Adult Intelligence Scale Vocabulary and Raven Matrices). They showed significant deficits in reading and writing and in reading-related phonological processes. Additionally, their processing rate was overall slower and their short-term memory spans were shorter. The students with dyslexia also had poorer arithmetic performance. Dyslexic students further reported more problems with memory (“I easily forget about what has been said”), attention (“I lose track in required reading”), effort (“I do not work to my potential”), affect (“I am sensitive to criticism”), and – less so – organizing and activating (“I have difficulty getting organized and started”). Based on these results, Hatcher et al. [14] doubted about the generality of the statement that higher education students with dyslexia have compensated for their problems.

Surprisingly, Hatcher et al.’s [14] rather pessimistic conclusion was not followed by other studies of the same standards. Subsequent studies again involved small numbers of tasks and small numbers of participants, making it difficult to obtain reliable estimates of the overall cognitive profile of dyslexic students in higher education [15]–[16]. A further step forward was made when Swanson and Hsieh [17] published the results of a meta-analysis. By applying such an analysis, researchers can distill a coherent pattern out of a multitude of heterogeneous, small-scale studies. Swanson and Hsieh’s meta-analysis was based on 52 published articles (but surprisingly without Hatcher et al. [14] and 776 comparisons of participants with reading disabilities versus participants without reading disabilities. An additional advantage of meta-analyses is that the results are communicated as effect-sizes. Swanson and Hsieh used Cohen’s d statistic. This is a standardized measure with very much the same interpretation as a z-score [18]. As a rule of thumb, d-values larger than.5 have practical value and d-values larger than.8 point to a substantial difference between the groups. These effect sizes make it easy to translate research findings to the counseling practice. In contrast, individual studies have a tendency to focus on the statistical significance of their effects, often overlooking issues of power and practical importance.

Table 1 summarizes the findings reported in the meta-analysis of Swanson and Hsieh [17] as effect sizes (d) of differences between participants with reading disabilities and participants without reading disabilities. Positive values indicate poorer performance of participants with dyslexia; negative values indicate better performance of this group. For comparison purposes, we also include the data of Hatcher et al. [14] expressed as effect sizes. The convergences between both studies are clear. The top problems of adults with dyslexia are, not surprisingly: writing, reading, and phonological processing (non-word naming and spoonerisms, which involve exchanging the first sounds of two words, e.g., turning “Terry Wogan” into “Werry Togan”). The effect sizes are mostly larger than 1. In addition, reading disabled adults seem to be poorer in retrieving verbal information from long-term memory, either because this information has been processed less frequently or because of an additional weakness in individuals with dyslexia. One of the most robust findings in cognitive psychology is the (word) frequency effect, the finding that the efficiency of information processing depends on the number of times the information has been processed before (e.g. [19]). There was also poorer performance on arithmetic. This finding has recently been confirmed [20]–[21] and linked to the fact that arithmetic operations often depend on verbal fact retrieval, in particular for multiplication. This would explain why the difference between individuals with dyslexia and controls is larger for multiplication than for subtraction [20].

**Table 1:** Effect sizes (d) of differences between participants with reading disabilities and participants without reading disabilities.

	S&H09	HSG02
**Literacy**		
Reading comprehension	1.20	
Word reading	1.37	1.14
Non-Word Reading	1.33	1.47
Word Spelling	1.57	1.31
Text Writing	0.72	1.12
Processing skills		
Perceptual speed		0.89
Short-term memory span		1.05
Phonological skills		
Phonological processing	0.87	1.32
Rapid naming	0.96	1.19
Verbal fluency		
Semantic fluency		0.46
Rhyme fluency		1.26
General intelligence		
Arithmetic	0.75	0.58
Verbal memory	0.20	
Verbal intelligence	0.63	
Vocabulary	0.71	0.10
General information	0.47	
Problem solving/reasoning	0.11	−0.01
Verbal memory	0.62	
Visuospatial memory	−0.39	
Cognitive monitoring	0.27	
Perceptual motor skills	−0.13	
Auditory perception	−0.18	
Visual perception	0.13	
Other		
Social and personal skills	0.10	
Personality	0.28	
Neuropsychological (e.g., EEG)	−0.02	
Ratings by third persons	−0.23	

*Note:* S&H09 =  Swanson & Hsieh [17]; HSG02 =  Hatcher, Snowling & Griffiths [14].

On the positive side, there were no differences of practical significance for general intelligence, problem solving/reasoning, cognitive monitoring, perceptuo-motor skills, auditory and visual perception, social and personal skills, personality, and neuropsychological measures (such as EEG patterns). Dyslexics slightly outperformed controls in visuo-spatial memory and tended to be rated more favorably by third persons than controls.

All in all, Swanson and Hsieh’s [17] analysis paints a rather clear picture of the strengths and weaknesses of adults with dyslexia. Still, two caveats should be kept in mind. The first is that meta-analyses involve a combination of very heterogeneous studies, with varying degrees of methodological rigor. This is particularly a concern when the number of studies on which an effect size has been calculated is rather small. Then, the presence or absence of an effect could be due to a single unrepresentative study involving a less valid test or a less representative participant sample. This issue is known as the apples-and-oranges problem in meta-analyses [22]. Although the convergence between Swanson and Hsieh [17] and Hatcher et al. [14] is reassuring in this respect, one would feel more confident if the picture were confirmed in an independent series of studies given to the same groups of participants. The second caveat with respect to Swanson and Hsieh’s [17] conclusions is that they are almost entirely based on English-speaking adults. Only 5% of the data were from non-English studies. Below we discuss two reasons why generalization to other languages/countries is not straightforward.

### Factors that may Prevent Generalization to other Languages

A first factor that may hinder the generalization of English findings to other languages, such as Dutch, is that languages differ in the difficulty of the letter-sound mappings. This feature has been linked to the time children need for reading acquisition [23]–[25] and also to the prevalence of dyslexia ([26]; see also [27] and [28] for a discussion of the ways in which English differs from other languages and what impact this may have for dyslexia). Readers of languages with inconsistent mappings need more time to reach ceiling performance and also have higher chances of not succeeding. There are two types of mapping: from letters to sounds and from sounds to letters (particularly important for correct spelling but also involved in word reading; [29]). Alphabetical languages differ in the degree of complexity of these mappings [30]–[31] with English consistently being the most opaque for both directions, and Dutch more towards the transparent end of the continuum (the extent depending on the specific measure used).

In the absence of empirical evidence, it is not clear what to expect as a result of the language differences in letter-sound mappings. On the one hand, one could imagine that dyslexia would be less of a problem in a transparent language; on the other hand, someone with dyslexia in a transparent language may on average have a stronger deficit than someone with dyslexia in an opaque language (if indeed differences in prevalence of dyslexia because of language transparency exist).

Another factor that may limit the findings of Table 1 to English-speaking countries is the organization of the education system in different countries. In general, British-inspired education is characterized by ability-based selection at the entry together with a commitment to bring the selected candidates to a successful completion (the master-apprentice model). In many other countries, however, there are no hard entrance criteria for higher education, and selection occurs as part of the curriculum. In Belgium, for instance, everyone who has completed secondary education, is entitled to start whatever type of higher education they want (except for medicine and dentistry, where an additional entrance exam must be passed). As a result, the number of students starting higher education tends to be higher and completion rates are lower. In particular, the first year is considered as a selection year with less than half of the student succeeding. Classes in the first year, therefore, tend to be plenary lectures before large groups, and exams often are multiple choice.

Needless to say, ability-based admission criteria are likely to have implications for the cognitive profiles of the students, certainly in the first year of education. For instance, the observation that Swanson and Hsieh [17] and Hatcher et al. [14] found no differences in general intelligence or problem solving between students with and without reading problems may be a consequence of the fact that British and American universities select their students on the basis of SAT-scores (US) and A-levels (UK). Indeed, Lovett and Sparks [32] noticed that a discrepancy between general intelligence and reading skills in American university students with reading disabilities is often due to average text reading skills combined with above-average IQ. Such a pattern might be a direct consequence of the admission criteria. As these criteria are not present in Belgium, students with quite different IQ-scores can start the same degree and there is no built-in guarantee that students who present themselves with a diagnosis of dyslexia have the same abilities as students without such an assessment. On the other hand, because students with a reading disability know of the selection taking place in the first year of higher education, they may be less inclined to start a degree that is perceived as demanding, given the chances of failure.

### The Cognitive Profile of Students with Dyslexia in Higher Education: Evidence from Non-English Speaking Countries

As stated before, literature on dyslexia in young adults who do not have English as mother tongue, is limited. In addition, in line with the first studies in English, they all focused on weaknesses rather than on the full pattern of strengths and weaknesses. Reid, Szczerbinski, Iskierka-Kasperek, and Hansen [33] ran a study in Polish on 15 dyslexic university students and 15 control students. As primary deficits they reported inferior word reading rate, pseudoword reading rate and text reading (both speed and accuracy). Spelling accuracy was also significantly lower. In relation to the underlying causes of dyslexia the authors observed impaired rapid automatized naming (pictures, colors, letters and digits) and phonological difficulties on a timed sound deletion task. However, group differences on spoonerism accuracy/time and sound deletion accuracy only approached significance. Similar results were found in a French study by Szenkovits and Ramus [34]. Students with dyslexia (N = 17) performed worse than a control group on a text reading task when a combined time and accuracy measure was reported (but see Bruyer and Brysbaert [35] for difficulties with such combined measures). Orthographic skills were also significantly lower. Moreover, a combined RAN (colors, digits and letters) score revealed impaired automatized naming and working memory. Students with dyslexia also displayed phonological deficits. Wolff [36] examined Swedish university students (N = 40) on a range of reading, writing and phonological skills tasks. Significant differences with large effect sizes were reported for several tasks: spoonerisms, non-word reading and writing (time and accuracy), exception word spelling, and orthographic skills (time and accuracy).

The above studies agree with the English studies showing that difficulties in reading and writing and phonological impairments persist into adulthood. However, none addressed abilities beyond reading and writing. Furthermore, they were all characterized by small sample sizes, making it dangerous to interpret the effect sizes.

### A New Study

Given the limitations of the available evidence, we decided to run a new study, which would enable us to compare the American-British profile (Table 1) to the Belgian profile. In order to do things properly, we took into account the following methodological considerations.

**Figure 1:**
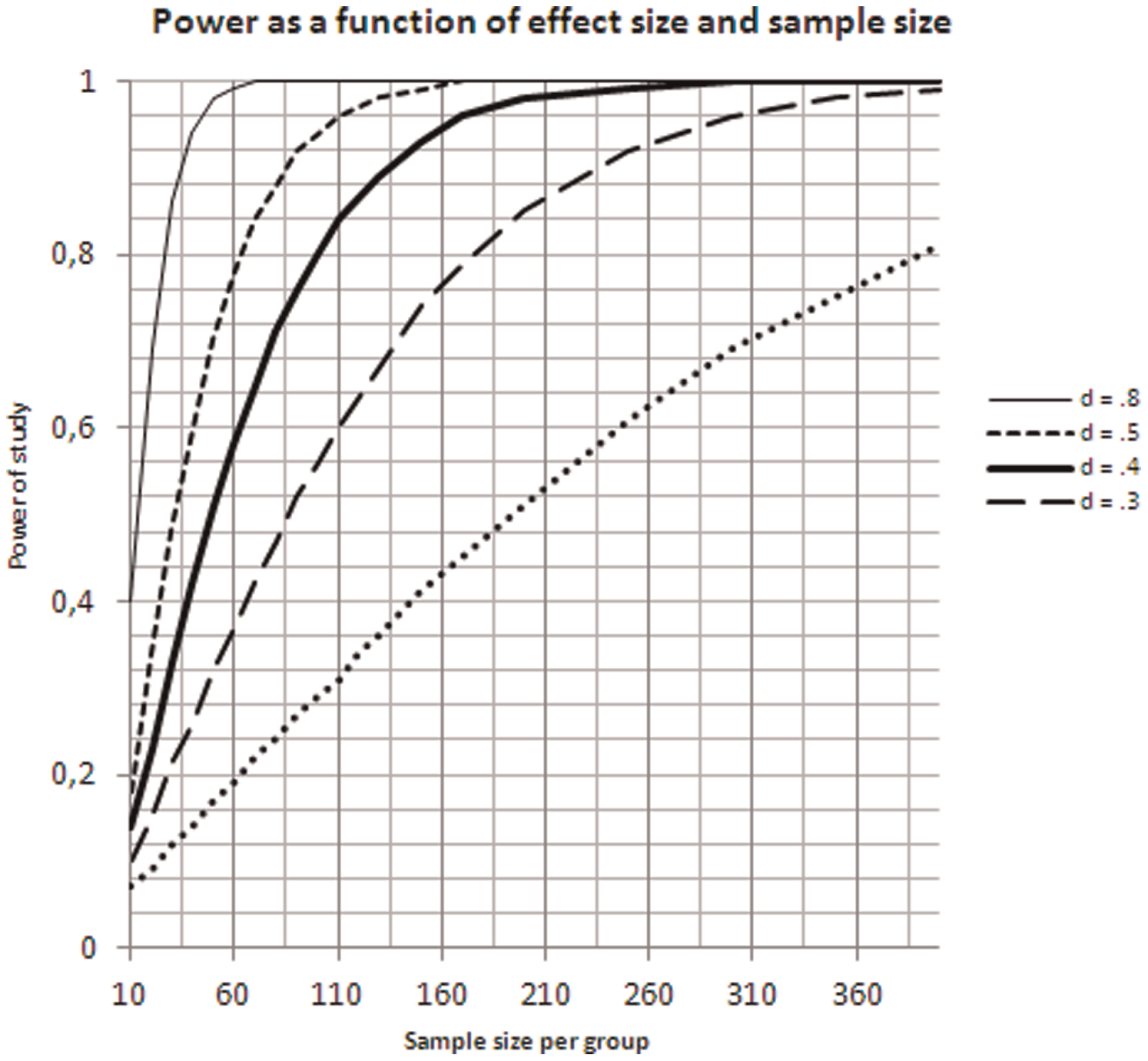
Power of study as a function of sample size. This figure shows the power of a study with two independent groups as a function of sample size for different levels of effect size (assuming that alpha, 2-tailed, is set at.05). For a small effect size (d = .2) we would need two samples of 393 participants to yield a power of 80%. This means that there is 80% chance of finding a significant difference between the groups, given that an effect of this size exists at the population level. For a medium effect size (d = .5) we would need two samples of 64 participants to achieve this level of power. For a large effect size (d = .8) we need 26 participants per group.

A problem with small-scale studies for applied research is the large confidence intervals around the obtained statistics, certainly in between-groups designs involving the comparison of two samples of individuals. Only recently have researchers become sensitive to the power problem related to small-group comparisons (e.g [37]–[38]). The smaller the samples, the larger the difference between the groups at population level must be before it can be found reliably in an empirical study. As a rule of thumb, to assess effect sizes as small as d = .4, one requires two groups of 100 participants (Figure 1). Samples of this size also result in reasonably small confidence intervals, so that the observed effect sizes can be trusted and compared to those from the English studies (Table 1).

To further improve the relevance of our study for offices of disability studies, we ran the study on the first 100 students who were entitled to special educational support on the basis of dyslexia by a learning disability support office in the city of Ghent (Belgium) and who were willing to take part in our study. For each student we then looked for a control student matched on age, gender, and field of study. The local support office serves Ghent University as well as other colleges of higher education (including technical colleges), meaning that we could examine a wide range of students.

## Methods

### Participants

Two hundred first-year undergraduate students of higher education participated in the study, both students of professional bachelors (in colleges for higher education) and academic bachelors (in some colleges for higher education and in university). They all attended higher education in Ghent, one of the major cities of Flanders (the Northern, Dutch-speaking half of Belgium) and had just graduated from secondary school. The group consisted of 100 students diagnosed with dyslexia and a control group of 100 students with no known neurological or functional deficiencies. All had normal or corrected-to normal vision and were native speakers of Dutch. Students were paid for their participation. The study followed the ethical protocol of Ghent University, meaning that students gave informed consent and were informed that they could stop at any time if they felt they were treated incorrectly.

The students with dyslexia had been diagnosed prior to our study by trained diagnosticians in accordance with the definition of SDN (Stichting Dyslexie Nederland [Foundation Dyslexia Netherlands] [39]). Because of the ongoing debate about the origin of dyslexia, the SDN uses a purely descriptive definition of dyslexia. In their guidelines dyslexia is defined as an impairment characterized by a persistent problem in learning to read and/or write words or in the automatization of reading and writing. The level of reading and/or writing has to be significantly lower than what can be expected based on the educational level and age of the individual. Finally, the resistance to instruction has to be confirmed by looking at the outcome of remedial teaching. Remedial teaching is considered adequate when it meets the requirements as stated in the “response to instruction” model [40] or the “response to intervention’ model [41]. Also, the SDN definition requires ensuring that the reading and writing impairment cannot be attributed to external and/or individual factors such as socio-economic status, cultural background or intelligence. Students entering higher education in Ghent are assessed anew by the local support office (*vzw Cursief*) if their previous assessment is older than three years. All students with dyslexia had (sub) clinical scores (<pc 10) on a word reading test [42] and/or, pseudo word reading test [43] and/or word spelling test [44]. These tests are addressed further in the text. All students with dyslexia had received individual tutoring in primary or secondary education for a period of minimum 6 months by either a speech-therapist or a remedial teacher.

All students with dyslexia who applied for special facilities at the local support office in the academic year 2009–2010 were asked to participate in the study until we had a total of one hundred. To find a group of 100 participants with dyslexia who completed the full study, we had to approach an initial cohort of some 120 students. Of these 120 students a small number of students chose not to cooperate once the study was explained to them. A few more students were lost because they failed to show up at appointments.

When recruiting the subjects we tried to reflect the inflow in the first year of higher education as much as possible. Matching criteria for the control students were therefore restricted to field of study, gender and age. Because one of the goals of our project is to see how dyslexic students succeed in higher education compared to their peers and to assess the impact of their disability on their study skills we matched them on field of study. We did add age and gender as matching criteria to construct homogenous groups. To recruit the control students we used different methods. We asked the students with dyslexia for several names of fellow classmates who would be interested in participating. Amongst these names we selected someone at random. In case the dyslexic student failed to deliver any names (which was the case for about 50% of the participants), we recruited them ourselves by means of electronic platforms or the guidance counselors of the institution in question. Table 2 contains the general information on the two groups: mean age, gender, professional bachelor v. academic bachelor students, fields of study and the educational level of the parents.

**Table 2:** General information on the control group and the group with dyslexia.

	Control group		Dyslexia group	
**Mean age**	19 years and 11 months		19 years and 4 months	
**Gender**	46 male students54 female students		46 male students54 male students	
**Degree taken**	66 non university college students34 university students		66 non university students34 university students	
**Field of study**				
	Non university students	University college students	Non university students	University college students
Educational sciencesHealth and behavioral sciencesManagementSciences and EngineeringArts and humanitiesOther	162191901	01901050	162191901	01901050
**Educational level father**				
Lower secondaryHigher secondaryCollegeUniversityMissingTotal	44428168100		73631224100	
**Educational level mother**				
Lower secondaryHigher secondaryCollegeUniversityMissingTotal	4364578100		43541182100	

The socio-economical level of the parents was not a matching criteria but no difference was found between the two groups in socio-economical level based on the educational level of the mother [χ^2^(3) = 4.855, p = .183] and father [χ^2^(3) = 2.634, p = .452]. Educational levels were: lower secondary education, higher secondary education, post secondary education either at university or a college for higher education.

### Cognitive Measures and Tests Administered

In the following section, we group the tests as a function of cognitive skill. Although this is not 100% how the assessment happened (which was battery-based), it makes it easier to compare our data to those of Swanson and Hsieh [17] and Hatcher and colleagues [14]. Most cognitive skills were assessed with validated and widely used Dutch-language screening instruments. We used the Dutch version of the Kaufman Adolescent and Adult Intelligence Test [45] and an established test battery for diagnosing dyslexia in young adults [44]. We tried to obtain converging evidence from a second test designed to measure the same skill when no data about reliability and validity were available. In particular, we compared the data to the IDAA or *Interactive Dyslexia Test Amsterdam-Antwerp*, a test battery that at the time of our testing was being normed and validated [46].

The American KAIT, developed in 1993 by A.S. Kaufman and N.L. Kaufman, was translated by Dekker, Dekker, and Mulder in 2004 and norms were collected on a standardization sample in the Netherlands and Flanders. The main goal of the KAIT is to evaluate analytic intelligence in individuals from 14 to 85 years. In our study the complete version was administered. It consists of 10 subtests categorized into two types of intelligence: fluid and crystallized intelligence. The crystallized scale consists of 4 subtests: Word Definitions, Double Meanings, Auditory Comprehension, and Famous People (for more information see below). It reflects how well a person has learned concepts and knowledge that are part of one’s cultural and scholar context. It is influenced by verbal conceptual development and education. The fluid intelligence scale gives an indication of the person’s potential and flexibility to solve new problems, either verbal or non-verbal. The 6 subtests are Symbol Learning, Logical Reasoning, Secret Codes, Block Patterns, Delayed Auditory Memory, and Delayed Symbol Learning (for more information see below). The combination of fluid and crystallized IQ results in a total IQ-score. All three scores have a mean of 100 and a standard deviation of 15 points. Psychometric information can be found in Table 3.

**Table 3:** Reliability and validity indices for the different subtests of the KAIT [45].

	Internal consistency Chronbach’s alphafor age groups 16–19	Test-retest reliability forage group 14–24	Content validity: correlationwith WAIS –R Total IQ scores
**CIQ**	.92	.80	.79
Definitions	.82	.81	
Double Meanings	.81	.72	
Auditory Comprehension	.81	.71	
Famous People	.76	.87	
**FIQ**	.93	.84	.76
Symbol Learning	.93	.85	
Logical Reasoning	.81	.66	
Secret Codes	.80	.61	
Block Patterns	.80	.82	
Delayed Auditory Comprehension	.55	.49	
Delayed Symbol Learning	.93	.81	
**TIQ**	.95	.89	.84

We used the KAIT instead of the Wechsler Adult Intelligence Scale III [47] to avoid retest effects. Many students with dyslexia had been tested previously with the WISC or the WAIS as part of their assessment. Other reasons for choosing the KAIT were the less rigorous time constraints, which we considered an advantage for students with learning disabilities, and the inclusion of two subtests of delayed memory, namely Delayed Symbol Learning and Delayed Auditory Memory. Both subtests are considered valid measures of long term memory capacities.

We also administered the GL&SCHR, a Dutch reading and writing test battery for (young) adults [44]. This test includes many of the tasks frequently administered in dyslexia assessment (e.g. [14]). There are three tests specifically designed to evaluate reading and writing skills, namely Word Spelling, Proofreading, and Text Reading (for more information see below). Seven additional tests focus on associated language deficits such as phonological processing, rapid naming, short term memory and working memory, morphology, and vocabulary (for more information see below). Information about reliability can be found in Table 4. For different subtests different methods were used, namely KR20, Guttman split-half, and a test-retest correlation.

**Table 4:** Reliability indices for the different subtests of the GL&SCHR [44].

	KR20	Guttman split half (γ)	test-retest
Text Reading		.77< r <.90	
Word Spelling (Word Spelling and Proofreading)		.69< r <.80	
Reading Comprehension		.61	
Morphology and Syntax		.65	
Short Term Memory		.54< r <.77	
Vocabulary	.90		
Phonological Awareness (Spoonerisms and Reversals)			.78< r <.90
Rapid naming			.62< r <.84

The IDAA or Interactive Dyslexia Test Amsterdam-Antwerp [46] is a new diagnostic instrument for the diagnosis of dyslexia in secondary school children and students in higher education. It is a test battery developed by The University of Amsterdam, Lessius College for Higher Education (Antwerp), and Muiswerk for the screening of young adults. It focuses on core skills of reading and writing such as automatized word recognition, decoding at lexical and sublexical level, and orthographic and phonological competence. The individual administration is fully computer controlled. The battery consists of six subtests. The first one is a questionnaire that assesses print exposure in Dutch and English. Next, phonological skills are evaluated with a reversal task where the participant has to state whether the second orally presented nonword is the reversal of the first (e.g. rol-lor). Then, four tests focus on orthographical skills : flash reading in Dutch, flash typing in Dutch, flash typing of nonwords in Dutch, and flash typing in English. In these tasks participants are presented with a word or nonword for 200 ms followed by a mask (###). Depending on the task the participant has to identify whether the target item was a word or nonword, or type in the word/nonword. As the names indicate, this is done both for Dutch and English. As this instrument is still in development and copyright protected, the results can only be used as validation criterion for other measures.

Finally, we also administered some standard tests for reading and calculation problems, used in the Dutch-speaking countries, such as word and nonword reading tests, and a standard arithmetic test. All in all, the following cognitive functions were assessed.

### Literacy

#### Text comprehension

In this test from the GL&SCHR, a text is presented in printed form and at the same time read out by the computer. Afterwards, the participant has to answer questions about the text. These questions rely on either literal comprehension or deductive knowledge.

#### Word reading

A classic word reading test in the Dutch-speaking countries is the *EMT* [One Minute Test] [42]. Parallel-form reliability ranges from.89 to.97 in various studies, whereas test-retest reliability lies between.82 and.92. For more psychometric information about the EMT we refer to the test’s manual. The list consists of 116 words of increasing difficulty printed in four columns. The participant has to read aloud as many words as possible in one minute trying to minimize reading errors. Raw scores are obtained for the total number of words read, the number of errors made, and the number of words read correctly.

#### English word reading

We also administered an English version of the EMT, namely the One Minute Test or OMT [48]. Validity and reliability data of the OMT have been collected by Kleijen, Steenbeek-Planting, and Verhoeven [49]. Test-retest reliability varies between 0.87 and 0.92. This test is in all aspects comparable to the Dutch EMT, except that English words are presented instead of Dutch ones.

#### Text reading

In this test from the GL&SCHR participants are asked to read aloud a Dutch text which becomes increasingly difficult. Substantial errors (e.g. addition/substitution/omission of letters, syllables and/or words) and time consuming errors (e.g. repeating a word/sentence, spelling a word aloud) are registered as well as the total reading time.

#### Silent text reading

The test that was used -“Hoe gevaarlijk is een Tekenbeet? [How Dangerous Can a Tick Be?]”- is part of a screening instrument published by Henneman, Kleijnen, and Smits [50]. It provides an indication of silent reading speed and the ability to retain information. There are no norms for Flanders. So, we made use of the raw scores. To obtain further information about the validity of the test, we looked at the correlation with the EMT word reading test in our sample. A Pearson correlation coefficient of.66 (N = 200) was found. The silent reading test works as follows. Participants are instructed to read a text of 1023 words in silence, taking into account that they will have to write a short summary afterwards. During reading participants have to indicate the word they just read when a signal is given after one, two, and three minutes. Afterwards, the average number of words read per minute is calculated. The written summary is evaluated based on measures of content, structure and syntax but the results of these analyses are beyond the scope of the present paper [50].

#### Nonword reading

The standard Dutch nonword reading test is *De Klepel* [43]. The parallel-forms correlation varies between.89 and.95. In various studies, the results of the Klepel correlate between.74 and.91 with those of the EMT. For more psychometric information about the Klepel we refer to the test’s manual. The test contains 116 nonwords that follow the Dutch grapheme-phoneme correspondence rules. Administration and scoring are identical to the EMT.

#### Word spelling

Word spelling was measured with two tests of the GL&SCHR: *Word Spelling* and *Proofreading.* In the Word Spelling test participants write down 30 words dictated by means of an audio file with a 3 seconds interval. Afterwards they are given the opportunity to correct their answers. Half of the words follow the Dutch spelling rules; the other half are exception words (involving inconsistent sound-letter mappings that must be memorized). Participants are also asked to rate how certain they feel about each answer (certain, almost certain, uncertain). There is a score for the number of correct responses, one for the number of words written during dictation (speed of writing), and one total weighted score where the certainty per item is taken into account. When a correct answer is given and the participant is certain, the weighted item score is 5. When the word is spelled correctly but the participant is uncertain the score is only 2. The difference between the raw score and the weighted score can be considered as a measure of meta-cognitive knowledge [51]–[52]. In the Proofreading test participants are given 20 sentences in which they have to correct possible spelling mistakes and rate their certainty per item. Two scores are given: one for the total number of correct responses and a weighted score (see Word Spelling).

#### English word spelling

Given the importance of English in higher education, we also included an English word dictation test. We used a standardized English test for word spelling: the *WRAT-III English Word Dictation* [53]. The internal consistency coefficients for the English age groups 17–18 and 19–24 are both.90. For more information on validity and reliability in English we refer to the manual. Because this test has not yet been validated for bilinguals with Dutch as mother tongue, we calculated the Pearson correlation with the English flash typing test of the IDAA (r = 0.72; N = 200). The test was administered according to the guidelines in the English manual. The examiner says a word, uses it in a significant context, and repeats the word. The participant writes it down. The test consists of 42 words.

#### Sentence dictation

Because higher education involves academic language, we also administered an advanced spelling test (AT-GSN [General Test for Advanced Spelling in Dutch]), developed and used at the University of Leuven [54]. This test has been used in a number of scientific studies [55]–[56]. Further information about the validity was obtained by correlating the scores with those of the Word Spelling test of the GL&SCHR (r = .79) and with the Dutch flash typing test of the IDAA (r = .70). The test consists of 12 paragraphs with exception words and challenging spelling rules (e.g. for the verbs). The correct use of capitals and punctuation marks is also taken into account. The score is the total number of errors made.

#### Morphology and syntax

In this subtest of the GL&SCHR 20 sentences are presented, in which the participant has to identify the syntactical or grammatical errors. The same principles as in the Proofreading test are applied. The total score gives the number of correct answers, whereas the weighted score takes into account the certainty of the participant about the answer given.

### Processing Skills

#### Speed of processing

To measure the participants’ speed of processing, we used the CDT or Cijfer Doorstreep Test [Digit Crossing Test] [57]. This is a standardized Dutch test to detect attentional deficits and measure the speed and accuracy of processing in a task of selective attention involving task-switching. It is one of the 23 tests of the DVMH [Differential Aptitude Tests for Middle and Higher Level], a test battery published in 2003 by Dekker and De Zeeuw [58]. This test battery was developed according to Carroll’s [59] Three Stratum Model in order to assess a large variety of cognitive skills such as verbal and numerical reasoning, attentional skills and language skills. The test – retest reliability scores vary between 0.79 and 0.95. The test can be administered from 14 years up to 80. There are 960 digits from 0 to 9 presented in 16 columns. Students have three minutes to underline as many fours and to cross out as many threes and sevens as possible. Scores for working pace (total numbers of items processed), concentration (total number of correct items), number of target errors, number of missed target digits and percentage of errors are obtained.

### Phonological Skills

#### Phonological processing

Phonological awareness was tested with 2 subtests from the GL&SCHR: *Spoonerisms* and *Reversals*. In the *Spoonerisms* test the first letters of two orally presented words must be switched (e.g., Harry Potter becomes Parry Hotter). Accuracy and speed (measured with a stop-watch) are measured. In the *Reversals* test participants have to judge if two spoken words are reversals or not (e.g. rac-car). Again, accuracy and speed (measured with a stop-watch) are measured.

#### Rapid naming

In the RAN test of the GL&SCHR participants are asked to rapidly name letters, digits, colors, or objects presented one-by-one on a computer screen (4 tests). The participant determines the pace by pressing the Enter button. Accuracy and speed are measured.

### General Intelligence

#### Arithmetic

We used the *Tempo Test Rekenen* (*TTR;* [60]), a Dutch standardized test for mental calculations. It is designed to examine the rate at which participants mentally perform simple mathematical operations (single and double digits). There are five lists, consisting of additions, subtractions, multiplications, divisions below 100, and a random sequence of all four operations. Participants are given one minute per list to solve as many problems as possible. The score per subtest is the number of items minus the number of errors made.

#### General intelligence

The scores for crystallized IQ, fluid IQ and total IQ of the KAIT give us measures of general intelligence.

#### Vocabulary

We used three tests to evaluate this language function: *Vocabulary* from the GL&SCHR and *Definitions* and *Double Meanings* from the KAIT. In the *Vocabulary* test participants are asked to find the low frequency word for which a definition is given (e.g., the Dutch equivalents of anonymous or simultaneous). In the *Definitions* test the participant has to find a word based on a number of letters given and a short description of the word (e.g., “A dark color :.r.n”). In the *Double Meanings* test the participant has to find a word that is in some way connected to two word pairs (e.g., the connection between biology-body and jail-lock is the target word *cell*).

#### General information

To obtain information about the participants’ non-verbal long-term memory, we used the *Famous People* test of the KAIT. In this test pictures of famous people are shown and participants have to name the person (e.g., Ghandi).

#### Problem solving/reasoning

Three subtests for fluid intelligence of the KAIT [45] were used to evaluate this cognitive skill: *Symbol Learning*, *Logical Reasoning*, and *Secret codes*. In the Symbol Learning test, the participant has to remember and reproduce series of symbols in different sentence-like combinations. In the Logical Reasoning test, information is given about the relative location of a number of items (people or animals). By logical reasoning the participant has to infer the location of a target item. In the Secret Codes test three or four items are given a unique code consisting of different parts. Based on these codes the participant has to infer which codes new target items should get.

### Memory

#### Short-term memory span

The GL&SCHR contains a short-term memory test for phonemes and non-verbal shapes (which must be drawn), and a test in which participants have to reproduce randomly presented series of letters or digits in ascending order. The participant is placed in front of a computer screen. After pressing the enter button the participant sees and hears a series of items presented one item per 2 seconds. At the end of each series the participant has to reproduce the items remembered. The number of items within a series increases steadily.

#### Verbal memory

The GL&SCHR contains a short-term memory test for objects. Administration is identical to the short term memory spans test of the GL&SCHR described in the previous section.

#### Auditory memory

The *Auditory Memory Test* of the KAIT is a delayed memory task in which questions have to be answered about a text that was read out at the beginning of the administration of the KAIT (see the Auditory Comprehension Test discussed below).

#### Visuo-spatial memory

Visual-spatial memory was tested with two subtests of the KAIT: *Delayed Symbol Learning*, and *Block Patterns*. The Delayed Symbol Learning test is a delayed retention task of the symbols used in the Symbol Learning test. In the Block Patterns test a yellow-black pattern has to be reproduced with cubes.

### Auditory Perception

The *Auditory Comprehension* test of the KAIT comprises the presentation of *s*hort audio fragments about which the experimenter asks content questions. The participant has to provide an answer.

### Procedure

The complete test protocol was administered during two sessions of about three hours each. The protocol was divided into two counterbalanced parts. The order of tests in part one and two was fixed and chosen to avoid succession of similar tests. There was a break halfway each session. If necessary, students could take additional breaks. Students with dyslexia started with part one or two according to an AB-design. Their control student always started with the same part. All tests were administered individually by three test administrators according to the manuals guidelines. The test administrators were the two first authors and a test psychologist. To standardize administration each administrator read the manuals of the tests, had a practice session, and followed three sessions of the starting administrator. Testing occurred in a quiet room with the test administrator seated in front of the student.

## Results

To improve comparison with Table 1, results are given as Cohen’s d effect sizes (derived from parametric or non-parametric tests, see below). In line with the English studies (Table 1), the sign of the d-values was adapted so that positive d-values represent better performance of the controls and negative values better performance of the students with dyslexia. All data were first checked on normality and equality of variance between groups (dyslexic group and control group). When the constraints for parametric statistics were satisfied, means were compared using a Student’s t-test. Otherwise, the data were analyzed with the non-parametric Mann-Whitney-U test and converted into the appropriate d-value by means of the equation given in Field ([61], p. 530 on how to transform a U-value into an r-statistic) and an equation to derive the d-value from the r-statistic. Values of the t-statistics and U-statistics are not given, as these can be calculated from the d-scores. Table 5 shows performances of students with dyslexia on literacy skills in comparison with their non-dyslexic peers. For variables that were analyzed using with a t-test, confidence intervals for the effect sizes could be calculated with the use of the ESCI-CIdelta program [62]. In Table 6 the results of phonological skills and processing skills are listed. In Table 7 results on general intelligence measures are reported.

**Table 5:** Performances of students with dyslexia on literacy skills in comparison with their non-dyslexic peers.

	Students with dyslexia		Students without dyslexia		Cohen’s d			p
	M1	SD1	M2	SD2	d	lower CI	upper CI	
**Text comprehension (GL&SCHR)**								
Number correct responses	19.38	5.05	21.59	4.4	0.47^b^			**
**Word reading (EMT)**								
Total number read words	79.08	14.32	101.33	10.6	1.87^b^			**
Number of errors	2.05	2.10	0.91	1.12	0.67^b^			**
Correctly read words	77.03	14.21	100.42	10.58	1.97^b^			**
Percentage of errors	2.63	2.77	0.90	1.08	0.88^b^			**
**English word reading (OMT)**								
Total number read words	71.18	10.72	84.99	9.49	1.36^a^	1.05	1.67	**
Number of errors	3.99	2.70	2.53	2.15	0.59^b^			**
Correctly read words	66.52	10.2	82.49	10.20	1.40^a^	1.09	1.71	**
Percentage of errors	5.64	3.98	3.07	2.71	0.75^b^			**
**Text reading (GL&SCHR)**								
Substantial errors	15.71	10.80	7.81	5.19	0.98^b^			**
Time consuming errors	14.29	8.72	9.17	4.91	0.64^b^			**
Reading time	311.14	51.97	258.53	25.26	1.29^a^	0.98	1.59	**
**Silent text reading (Tekenbeet)**								
Words per minute	184.63	59.25	243.64	57.59	1.13^b^			**
**Nonword reading (Klepel)**								
Total number read words	46.07	9.84	63.26	12.90	1.50^b^			**
Number of errors	5.20	3.77	3.67	3.10	0.44^b^			**
Correctly read words	40.88	10.46	59.72	13.10	1.59^b^			**
Percentage of errors	11.75	9.11	6.05	5.28	0.88^b^			**
**Word spelling**								
Word Spelling								
Weighted score word spelling	91.59	15.87	121.40	12.84	2.28^b^			**
Correct word spelling	17.49	4.02	24.60	2.81	2.05^b^			**
Writing speed	24.89	4.01	26.50	3.40	0.43^a^	0.15	0.71	**
Proofreading	51.23	10.96	63.49	11.69	1.08^a^	0.78	1.38	**
**English word spelling (WRAT)**								
Correctly spelled words	16.57	4.81	24.27	5.42	1.50^a^	1.19	1.82	**
**Sentence dictation (AT-GSN)**								
number of errors	54.04	24.17	23.20	11.65	2.10^b^			**
**Morphology and syntax (GL&SCHR)**								
Weighted score	50.34	10.35	59.57	9.86	0.91^a^	0.62	1.2	**
Total score	9.06	2.64	11.24	9.06	0.87^b^			**

p<.05; **p<.01.

*Note:* Parametric test results are marked with a. When the data violated the constraints for a parametric test, results are marked with b.

GL&SCHR  =  Dutch reading and writing test battery for (young) adults; EMT  =  Een Minuut Test [One Minute Test]; OMT  =  One Minute Test;

WRAT  =  Wide range Achievement Test; AT-GSN  =  Algemene Test- Gevorderde Spelling Nederlands [General Test Advanced Spelling Dutch].

With respect to the literacy skills (Table 5), the following results stand out:

1. As in English speaking individuals, the deficiency of students with dyslexia tends to be worse in the writing tests than in the reading tests. In particular, the Word Spelling test of the GL&SCHR and the Sentence Dictation (AT-GSN) resulted in large effect sizes (d ≈ 2).
2. Deficiencies in spelling are similar at the word level (d = 2) and at the sentence level (d = 2.1).
3. Dutch word reading (d = 1.97) seems to be more affected in students with dyslexia than nonword reading (d = 1.57), possibly because the former involved more instances of inconsistent spelling-sound mappings.
4. For our group of students in higher education deficiencies in reading and writing are not more pronounced in a second language (English) than in the first language. In English word reading the same pattern in effect sizes was found for the percentage of errors and the number of words read as in Dutch.
5. Reading deficiencies are most pronounced in speed (1.60< d <1.90). Smaller but still substantial effect sizes were found for percentage of number of errors made (d ≈.80).
6. Text comprehension was nearly equivalent for both groups (d = .4) when the text was read aloud, and better than expected on the basis of the reading scores.

Turning to the wider cognitive skills (Table 6 and 7), the following are the most important findings:

1. The differences on the IQ test are negligible and particularly caused by definitions to words (d = .75), although there is also a small difference for the recognition of famous persons (d = .35). There are no differences in fluid intelligence (d = .1).
2. Students with dyslexia tend to be slower than controls in processing speed as measured with the CDT(d = .6), and a small effect size can be noted for the percentage of errors (d = .35).
3. Except for phonological short-term memory (d = .71), memory spans are quite comparable (0.28< d <.45).
4. There is considerable dyslexia cost for arithmetic (d≈1), which tends to be larger for divisions (d = 1) and multiplications (d = .90) than for subtractions (d = .61).
5. There is a non-negligible cost (d >1.3) for phonological processing. This cost again is largely due to the speed of processing, and less to the accuracy of processing.
6. Dyslexics are slower at naming letters, digits and colors, but not at naming objects (d = .2).

**Table 6:** Performances of students with dyslexia on phonological skills and processing skills in comparison with their non-dyslexic peers.

	Students with dyslexia		Students without dyslexia		Cohen's d			p
	M1	SD1	M2	SD2	d	lower CI	upper CI	
**Processing skills**								
**Speed of processing (CDT)**								
Working pace	421.94	84.63	467.80	79.99	0.62^b^			**
Concentration	119.25	22.85	134.29	22.03	0.51^b^			**
Number of errors	0.19	0.56	0.15	1.73	0.23^b^			
Number of missed digits	8.08	6.96	6.60	6.76	0.19^b^			
Percentage of errors/missed	2.03	1.49	1.60	1.51	0.35^b^			*
**Phonological skills**								
**Spoonersims (GL&SCHR)**								
Number correct responses	16.72	2.50	18.19	1.67	0.70^b^			**
Time	179.88	65.98	116.48	41.22	1.42^b^			**
**Reversals (GL&SCHR)**								
Number correct responses	15.63	2.41	17.72	2.03	1.00^b^			**
Time	106.00	33.996	76.61	16.18	1.3^b^			**
**Rapid naming (GL&SCHR)**								
Letters	25.72	5.85	20.62	3.99	1.02^b^			**
Digits	23.83	5.26	19.28	3.64	1.05^b^			**
Colours	32.55	6.03	28.25	4.314	0.81^b^			**
Objects	39.55	7.39	37.84	6.82	0.24^b^			

p<.05; **p<.01.

*Note:* Parametric test results are marked with a. When the data violated the constraints for a parametric test, results are marked with b.

CDT  =  Digit Crossing Test [Cijfer Doorstreep Test]. GL&SCHR  =  Dutch reading and writing test battery for (young) adults.

**Table 7:** Performances of students with dyslexia on general intelligence in comparison with their non-dyslexic peers.

	Students with dyslexia		Students without dyslexia		Cohen’s d			p
	M1	SD1	M2	SD2	d	lower CI	upper CI	
**General Intelligence**								
**Arithmetic (TTR)**								
Total number calculations	121.24	20.67	144.75	23.83	1.05^a^	0.76	1.35	**
Addition	30.46	3.51	33.81	3.41	0.97^a^	0.67	1.26	**
Subtraction	27.31	4.17	30.14	3.98	0.61^b^			**
Multiplication	21.74	5.02	26.78	6.19	0.90^b^			**
Division	19.73	5.82	26.29	7.27	1.00^b^			**
Mixed operations	22.93	4.45	28.33	4.98	1.12^b^			**
**General Intelligence (KAIT)**								
Total IQ	105.50	12.97	109.83	9.29	0.38^a^	0.1	0.66	**
Crystallized IQ	106.66	8.11	111.31	8.83	0.55^a^	0.27	0.83	**
Fluid IQ	105.36	11.04	106.78	10.83	0.13^a^	−0.14	0.41	
**Vocabulary**								
Vocabulary (GL&SCHR)	7.83	4.14	10.83	4.77	0.67^b^			**
Definitions (KAIT)	20.89	1.92	22.16	1.98	0.75^b^			**
Double meanings (KAIT)	14.44	3.91	16.10	3.71	0.43^b^			**
**General information (KAIT)**	7.26	3.14	8.41	3.25	0.35^b^			*
**Problem Solving/Reasoning (KAIT)**								
Symbol learning	80.45	12.64	80.93	13.14	0.07^b^			
Logical reasoning	11.32	3.48	11.78	3.18	0.12^b^			
Secret codes	26.78	5.49	27.46	4.91	−0.13^b^			
**Memory**								
Short term memory span (GL&SCHR)								
STM phonemes	20.11	4.7	23.23	4.56	0.71^b^			**
STM shapes	10.44	4.00	11.84	5.05	0.28^b^			*
Memory with sorting	39.34	5.03	41.54	4.34	0.45^b^			**
Verbal memory (GL&SCHR)								
STM words	35.41	5.78	37.24	5.37	0.30^a^	0.05	0.61	*
Auditory memory (KAIT)	4.99	1.40	5.54	1.50	0.37^b^			**
Visual Memory (KAIT)								
Delayed Symbol Learning	50.98	10.4	51.34	10.53	0.03^a^	−0.23	0.32	
Block Patterns	12.23	2.71	11.71	2.97	−0.17^b^			
**Auditory Perception (KAIT)**								
Auditory comprehension	13.26	2.96	13.60	2.80	0.09^b^			

* p<.05; **p<.01.

*Note:* EMT  =  Een Minuut Test [One Minute Test]; GL&SCHR  =  Dutch reading and writing test battery for (young) adults; AT-GSN  =  Algemene.

Test- Gevorderde Spelling Nederlands [General Test Advanced Spelling Dutch]; CDT  =  Cijfer Doorstreep Test [Digit Crossing Test]; TTR  =  Tempo.

Test Rekenen [Speed Test Mental Calculations], KAIT  =  Kaufmann Adult Intelligence Test; STM  =  short term memory.

Finally, to facilitate comparison with English, Table 8 includes our results together with those of Swanson and Hsieh [17] and Hatcher et al. [14]. In particular, the correspondence with Swanson and Hsieh is impressive. The Pearson correlation between both sets is r = .94 (N = 11, p<.001). The correlation with Swanson and Hsieh is lower if we also include the text comprehension difference of the present study (d = .5) and correlate it with the reading comprehension difference reported by Swanson and Hsieh (d = 1.2). Then the correlations drops to r = .74 (N = 12). However, this comparison is not really justified, because in our text comprehension test the text was additionally read out by the computer. Correlation is lower with Hatcher and colleagues [14], partly because of a lack of data in that study on aspects where students with dyslexia show good performance. The correlation coefficient is.67 and reaches significance (p<.05).

**Table 8:** Correspondence between the effect sizes reported in English and the effect sizes found in the current study.

	S&H09	HSG02	Dutch
**Literacy**			
Reading comprehension	1.2		
Word reading	1.4	1.1	2.0 (EMT correctly read)
Non-Word Reading	1.3	1.5	1.6 (Klepel correctly read)
Word Spelling	1.6	1.3	2.0 (GL&SCHR, N correct)
Text Writing	0.7	1.1	
Sentence dictation			2.0 (AT-GSN)
**Processing skills**			
Perceptual speed		0.9	0.6 (CDT Time)
**Phonological skills**			
Phonological processing	0.9	1.3	1.4 (GL&SCHR time)
Rapid naming	1.0	1.2	1.0 (GL&SCHR, without objects)
**General intelligence**			
Arithmetic	0.7	0.6	1.0 (TTR)
Verbal memory	0.2	1.1	0.3 (GL&SCHR, STM words)
General intelligence	0.2		0.4 (KAIT)
Vocabulary	0.7	0.1	0.6 (KAIT, GL&SCHR)
Problem solving/reasoning	0.1	−0.01	0.1 (KAIT fluid)
Auditory perception	−0.2		0.1 (KAIT, aud.compr)

*Note:* S&H09 =  Swanson & Hsieh [17]; HSG02 =  Hatcher, Snowling & Griffiths [14].EMT  =  Een Minuut Test [One Minute Test]; GL&SCHR  =  Dutch.

reading and writing test battery for (young) Adults; AT-GSN  =  Algemene Test- Gevorderde Spelling Nederlands [General Test Advanced Spelling.

Dutch]; CDT  =  Cijfer Doorstreep Test [Digit Crossing Test]; TTR  =  Tempo Test Rekenen [Speed Test Mental Calculations], KAIT  =  Kaufmann Adult.

Intelligence Test; STM  =  short term memory.

## Discussion

We designed this study to obtain an empirically based cognitive profile of students with dyslexia in higher education in a language other than English. We started from the tests we thought worthwhile, making sure those of Hatcher et al. [14] were included. Shortly after data collection began, Swanson and Hsieh [17] published their meta-analysis, providing us with an even more complete image of English-speaking students.

Despite the differences in language and educational context, our findings are remarkably similar to those in English: The pattern of strengths and weaknesses of students with reading disabilities is very much the same in Dutch as in English (Table 8). This is good news, because it means that the profile is likely to be applicable to all alphabetical languages. Also, different educational systems do not seem to play an important role in defining which students with dyslexia enter higher education.

A further important conclusion from our findings is that the data agree very well with the traditional definition of dyslexia as a combination of normal intelligence with deficient reading and writing. This definition has been questioned in recent years, because it has proven difficult to find the discrepancy in all individuals. Researchers have disagreed about whether this has theoretical consequences for the relationship between reading/writing skills and other abilities, or whether it is simply a consequence of the notoriously low correlations one is bound to find for difference scores of highly correlated variables (e.g. [63]). Our data leave little doubt that, as a group, dyslexics entering higher education show exactly the profile predicted by the traditional definition of dyslexia, even though at an individual level the difference scores may show large variability. As such, our findings reinforce a similar, tentative conclusion reached by Swanson and Hsieh [17].

The affirmation of the traditional definition of dyslexia shows that some lecturers’ doubts about the existence of isolated reading disabilities in combination with normal intelligence are unjustified. For the group we tested, we found – just like the authors before us – a pattern of results that is extremely hard to obtain on the basis of deficient general abilities, motivation, or outright malingering. Although we cannot exclude the possibility that one or two of the students who refused to take part in our study did so because they wanted to play the system, our results emphatically testify that the vast majority of students entering higher education with a diagnosis of dyslexia are the same as the other students, except for a language-related deficiency that arguably hurts them most during the school years when they have to rapidly acquire and produce a lot of new information in written form.

At the same time, it is important to keep in mind that, although the differences are not large, all test scores tended to be lower for the students with dyslexia than for the controls. When looking at the full cognitive profile of students with dyslexia, it cannot be denied that there is a quite consistent deficiency on a wide range of tasks, predominantly those involving speed of processing and retrieval of verbal information from long term memory. It would be good if students with dyslexia were properly informed about this extra challenge they are facing. The most prominent example of such a “hidden” cost is the extra time they need for mental calculations (total of operations: d = 1), arguably because of the extra effort to retrieve arithmetic facts from memory (see the triple code model [64]). This additional deficit was not mentioned by many students, but is likely to cause problems in courses involving the calculation of many elementary arithmetic operations (e.g., the calculation of a standard deviation in a course of statistics).

Sometimes it has been hypothesized that successful individuals with dyslexia have fully compensated for their reading and writing difficulties [65]. Hatcher et al. [14] raised doubts about this possibility, and our data confirm this to some extent, although the picture is much less pessimistic. What is encouraging is the finding that students with dyslexia tended to perform equally good on the text comprehension test, in which the text was additionally read out by the computer (see also their good scores on the auditory comprehension test). This suggests the usefulness of text-to-speech arrangements, although ideally we would have more data on this aspect, directly comparing text comprehension with and without text-to-speech assistance.

A further interesting finding of our study is that the effect sizes are not larger for tests based on sentences than for tests based on individual words (word reading d = 1.87, text reading d = 1.29; word writing d = 2.05, text writing d = 2.10). This agrees with the descriptive definition of SDN [39] arguing that the impairment in reading and spelling can be measured at the word level. Our data indicate that tests of reading and writing at the word level are enough to make a valid diagnosis. This is valuable information for diagnosticians, as it leads to a substantial time gain.

Finally, our findings have clear implications for guidelines about special arrangements. We think the following arrangements are incontestable:

1. It is clear that students with dyslexia have a specific and pervasive problem with reading and writing. This means that they are entitled to arrangements that help them with these particular deficiencies, such as text-to-speech software (also during exams) and the use of spellcheckers and word completion software when spelling errors are likely to lead to lower marks (e.g., for essay-type questions).
2. Students with dyslexia are at a disadvantage under time constraints, meaning that situations should be avoided in which they are likely to suffer more (e.g., exams and tests with strict time limits). This does not mean that students with reading disabilities should be given extended deadlines for all tasks (e.g., for the submission of essays and lab reports, which can be planned well in advance), but it does entail that they are denied a fair chance if they have to complete an exam in the same time as their peers.
3. Many students with dyslexia have a pervasive problem with mathematical tables. This should be taken into account when an exam strongly relies on them (e.g., for problem solving, where different alternatives have to be tried out). This problem can easily be solved by allowing students to use a calculator.
4. Finally, there is scope for better feedback to the students themselves. It is important for them to know of the limitations they are confronted with, so that they can prepare themselves well and insist on having the arrangements outlined above. A better knowledge of their limitations may also help them not to overestimate their abilities. One cannot deny that the average performance of the dyslexics on nearly all tests tended to be lower than that of controls. Although these differences often are too small to justify special arrangements, students with reading disability should know about these differences, so that they can better organize their studies. For instance, many institutes of higher education nowadays provide their students with ways to spread the burden (e.g., by studying part-time or distributing the exams over extra sessions). It may be an idea to discuss these options with students (and their parents), certainly when their test performances are below average, so that they can prepare themselves better in the light of the specific difficulties they will be confronted with.

The above (minimal) arrangements are easy to implement if they are part of the general organization of exams, certainly with the current availability of text-to-speech software and text writing software with built-in spellcheckers. Additionally, these measures are so specifically tailored to the proven needs of students with dyslexia that they are unlikely to be contested or misused. To our knowledge there is no evidence that text-to-speech software, spellcheckers, and a few extra hours for exams are any good in compensating for a lack of knowledge, deficient intellectual abilities, or missing achievement motivation. However, our results strongly suggest that they will make a significant difference for students with dyslexia.
